# Hospital and Institutional News

**Published:** 1917-07-21

**Authors:** 


### HOSPITAL AND INSTITUTIONAL NEWS.

### THE ROYAL COLLEGE OF SURGEONS.

We congratulate the Royal College of Surgeons on the election of Sir George Henry Makins, K.C.M.G., C.B., as its President. Few, if any, men in the profession have done such continuous and splendid work as Sir George Makins has accomplished since he took the Fellowship in 1878. In thirty-nine years he has rendered noteworthy service to the profession in connection with the International Medical Congress in London, where his administrative and literary gifts were so well applied as to make the Congress with which he was so closely identified, memorable, both in regard to their organisation and to the splendid efficiency with which the reports of the proceedings were produced and published in record time. His services to St. Thomas's Hospital have given him a world-wide reputation as a surgeon. They have won for him the admiration and affection of his colleagues and the governors of St. Thomas's Hospital, who, on his reaching the age of retirement, granted him an extension for two years of his office of surgeon to the hospital, and subsequently invited him to continue on the staff at least until the termination of the war. Sir George Makins' work in connection with the war as consulting surgeon to the Expeditionary Force in France, and the active and continuous labour involved, coupled with his splendid services to the nation, will ensure for them a notable place in its history. The famous American surgeon, Mr. William Mayo, in one of his books records the opinion that to watch Sir George Makins' work and technique in the operating theatre made him feel that, should occasion arise, Sir George was the man he would desire to act as surgeon to himself and to members of his family. Our soldiers at the Front have indeed been fortunate to have Sir George Makins as their consulting surgeon. An intimate knowledge of what Sir George has accomplished enables us to endorse and emphasise the precise accuracy and justice of the opinions here given.

### COLONEL CARTER AND SIR VICTOR HORSLEY.

Last week we reproduced from the *Weekly Dispatch* a portion of the late Sir Victor Horsley's letter to Major (now Lieut.-Colonel) Carter. Sir Victor Horsley's letter somehow came into the possession of the *Weekly Dispatch*, which published it in full on July 8. The *Weekly Dispatch* declares in its issue of the 15th instant that it published this letter without the consent or knowledge of Lieut.-Colonel Carter, to whom it was addressed. It is a matter of some importance to know by what means our contemporary obtained this letter, which presumably came before the Commission and so passed through various official departments. Was a copy of Sir Victor Horsley's letter made by someone through whose hands it may have passed? If so, who was this person and did money pass in so discreditable a transaction? It is our privilege to-day to publish these circumstances, which exonerate Lieut.-Colonel Carter from a possible charge of deliberate self-advertisement through this publication of Sir Victor Horsley's letter. Parliament has been occupied during two whole days in debating the report of the Commission on Mesopotamia. The country and the profession are uneasy from the feeling that no adequate punishment, if indeed any punishment at all, will be administered, if politicians can prevent it, to those in authority or otherwise who caused or contributed to the unnecessary and very heavy loss of life in Mesopotamia, and to the avoidable suffering of the wounded, which was awful; nor for the gross and persistent mismanagement of the Medical Services, detailed at great length in the Vincent-Bingley Report presented prior to that of the Mesopotamia Commission. There is reason to fear there may be some who hope that Lieut.-Colonel Carter has been pushed into a useful and restful obscurity, where he will be rewarded for his courageous and patriotic fight on behalf of our warriors in Mesopotamia by losing nearly everything, because of his brave attempt to do his duty, despite the threats of some who have yet to be dealt with. Such a possibility, however, is unthinkable, and constitutes a danger which neither the King, the Government, nor the nation can suffer.

### FRANCE'S DAY.

We have had many "days," and there are no doubt many "days" to come, but none have been, or will be, more popular than "France's Day." Not many members of the British public have any but a vague idea of what the taking of the Bastille meant in its effect on French history, but an occasion, whatever the anniversary, which afforded an opportunity of helping an ally of Britain that has suffered so severely as France could not be passed by without ready response by the British public. Apart from sentiment the French Red Cross is intrinsically worthy of generous support, its hospitals, as the French Ambassador says in his message of thanks, are models of efficiency; its ambulance, nursing, and other services are an important part of the military organisation. It was fitting that the celebration of July 14 should open with a solemn requiem for France's fallen heroes, celebrated by the Cardinal-Archbishop of Westminster at the Roman Catholic Cathedral, and attended by M. Paul Cambon and his staff. For the street collections no fewer than 20,000,000 souvenirs were provided, and the general impression at the close of the day was that very few of them were left unsold. According to the estimate of the French Red Cross Committee, a sum of not less than £250,000 is required to provide for the efficient continuation of its work, which is now under an additional and growing burden in the relief of refugees and of the ravaged districts. A very substantial contribution towards this important sum must have been made as a result of France's Day.

### THE RED CROSS IN SCOTLAND.

On July 2 a special six days' effort was commenced throughout the length and breadth of Scotland to obtain additional funds for maintaining and extending Red Cross work. In Glasgow preparations on a most lavish scale were made, so as to attract all sorts and conditions of people. The Royal and Stock Exchanges, public works, shopkeepers, banks, hotels, picture-houses, and restaurants, all lent a helping hand. Edinburgh played its part in the movement right well. The inevitable Flag Day was made use of, and was most good-humouredly supported by the public, who purchased over 350,000 souvenirs. Theatrical people came to the aid of the fund, and a special matinée performance of the revue "Zigzag" was given at the Empire Theatre, lent free by the Moss Empires, Ltd., while at the Gaiety Theatre, Leith, a crowded house supported a grand concert organised for the occasion. On Sunday the churches had their turn, and special collections were made throughout the city. One of the prettiest features was the children's display on the Saturday in Princes Street Gardens, combining dancing and musical interludes, while the Newhaven Fisher Girls acted as collectors, their picturesque costumes attracting many subscribers. Altogether, thanks to thousands of willing workers, Scotland's Red Cross Week exceeded all expectations, and it is confidently estimated that the total sum aimed at, £100,000, has not only been reached but exceeded.

### MEDICAL SERVICES IN MESOPOTAMIA.

General Sir Stanley Maude's dispatch from Mesopotamia—the clearest and best-written military dispatch since General Sir Ian Hamilton's on the Gallipoli operations—shows that the Medical Service there has now been placed on a sound and efficient footing. In view of the scandalous state of things which previously existed on the Tigris in relation to the treatment of the wounded, General Maude's tribute to hospital efficiency is of more than ordinary interest, and is welcome reading to the people of this country. "Our well-equipped hospitals," he says, "have been more than adequate to meet the calls made upon them by sick patients. Throughout the operations the evacuation of the wounded was carried out on model lines, and the arrangements made for the comfort and rapid transfer of patients from the field units to the hospitals on the lines of communication reflect much credit on those concerned. Whilst those on the lines of communication have done their share efficiently, the work of the Medical Services at the Front has maintained its high reputation. During the operations the strain thrown upon all has been heavy, and the courage and devotion shown by the *personnel* on the battlefields has only been equalled by the zeal and energy of those in the field units." Sir Stanley Maude speaks highly of the valuable services rendered by the consulting surgeons and physicians, and adds, "The thanks of the Army are due to the nursing sisters for their indefatigable services in tending the sick and wounded. These ladies have, by their devoted work under difficult conditions of climate and surroundings, set an example of which they may well be proud."

### MEDICAL EDUCATION FOR INDIA.

The hideous revelations of the Mesopotamia Report have once again drawn public attention to the problem of providing in India adequate instruction for native medical students who do not come to England for their degrees. The Report of the Islington Commission on Public Services in India is already two years old, and little has apparently been done to guarantee that "even under the gravest war conditions the civil cadres shall not be unduly depleted, and in particular that no dislocation of the educational and scientific work of the country shall take place." They recommended that the various special departments—the professorships and chemical examinerships, with their connected posts, and the alienist department should be formed into separate units, and the officers holding them should be excluded altogether from the liability of recall to military duty. Again, of the thirty-seven whole-time professorships in Government medical colleges no fewer than thirty-three are ordinarily filled by members of the I.M.S. It would seem, in view of the fact that the competition for these posts is necessarily limited, that the public interests would be better served by throwing them open to civilian candidates. The Commission looks forward to the time when the teaching in preliminary scientific subjects can be arranged for through the various universities. It is certain that if we are to be spared a repetition of the Mesopotamia scandals the I.M.S. will have to be remodelled, both from a financial and from an educational standpoint.

### REMEDIAL BATHS FOR ORTHOPÆDIC CASES.

The new department, given to King Edward VII.'s Hospital, Cardiff, in memory of his wife, by Sir John Gunn, for the treatment of orthopædic cases by remedial baths, was opened recently by Dr. R. Fortescue Fox. He declared that the Lady Gunn Hall, as the new department is called, was an improvement on anything he had seen in other places. It was most important to send back wounded men completely cured, and this treatment by remedial baths was one result of the research to which the new School of Medicine for Wales was committed. Colonel E. M. Bruce Vaughan, in moving a vote of thanks to Sir John Gunn, expressed the opinion that a chair of climatology and hydrology should be established at Cardiff, or if not a chair, at least a lectureship. Sir John Gunn acknowledged the vote of thanks by saying that he was merely one of the instruments Colonel Bruce Vaughan knew so well how to employ in bringing the hospital up to date.

### HEALTH OF THE SALONIKA ARMY.

It is more than an open secret, if it ever was a secret at all, that last year the health of the troops in Salonika and its neighbourhood was extremely bad, a very large proportion suffering from malaria, dysentery, typhoid, and similar maladies. It is, therefore, especially gratifying to learn that a great improvement has taken place, and this year compares very favourably with 1916. For instance, during the four weeks ending June 23, 1916, the number of cases of sickness per 1,000 men was for malaria 7.79, while this year the number has been reduced to 5.65. In the same way the number of cases of dysentery per 1,000 men last year was over twenty, and this year the number is under seven. Last year the cases of typhoid were very few in number, only .37 per 1,000 men, a very eloquent testimony to the efficacy of typhoid inoculation; yet this year the number of cases has been reduced even below this, for there have been only .02 cases per 1,000 men. This improvement is extremely satisfactory, especially in connection with the fact that, as we understand, it has been resolved to transfer some of the hospitals now in Malta to the neighbourhood of Salonika; not, indeed, in the city itself, but on higher ground where the hygienic conditions are much more favourable, and where the question of drainage can be much more easily solved.

### THE MATERNITY UNIT AT CARDIFF.

The establishment of a maternity ward and a home for children, which Sir Edward Nicholl's offer of £50,000 to King Edward VII.'s Hospital, Cardiff, made possible, has reached a step nearer completion with the effort made to provide an endowment fund. The provision of such a fund was the last of three conditions on which Sir Edward's offer depended. The first, that there should be no religious or other qualification for admission to the institution, was readily granted by the committee, and the second, that a freehold site be provided free of charge, has been fulfilled through the generosity of Lord Tredegar, who has given two acres for the purpose. On this land, which is a fine site, three homes will be built, one for boys, another for girls, and a third for infants, who will be received from birth onwards. At a special meeting recently held at the City Hall, Cardiff, it was decided to assist in raising an endowment fund by organising entertainments and collecting subscriptions. The sum of £20,000 is needed for maintenance, and the Church of England Waifs and Strays Society is interesting itself in the matter. The children of fallen sailors and soldiers will be admitted, and babies from birth whose parents are unable to look after them. The present effort was the last of Baby Week activities, and with the buildings and site secured, the endowment fund should surely be forthcoming.

### WHAT IS DISTRESS DUE TO THE WAR?

The York Citizens' Committee is the possessor of some £15,500 which has been subscribed for the relief of distress arising out of the war. There happens, as is usual with these funds, to be a considerable balance in hand, and advantage of this has been taken by an enterprising rescue home in the diocese to appeal for a grant from the committee's funds. The gift of a grant was declined on the ground that it was not the province of the Fund to subscribe to other institutions. At a recent meeting of the Fund this decision was canvassed, and finally a sub-committee was entrusted with the task of reporting on the scope of the committee's work in regard to distress arising out of the war, whether in the case of individuals or institutions. It was pointed out that to define what was distress arising out of the war was practically impossible, and also that if institutions were assisted the applications would be a lengthening list indeed. A warning was uttered in respect of the personal claims which would arise on demobilisation. These arguments are enough, in our view, to show that in principle the committee should confine its grants to individuals, that it should leave the determination of what actually constitutes distress due to the war to its own discretion, and that it should keep a certain proportion of its funds for those who will certainly need help immediately peace is concluded.

### THE PHYSIQUE OF THE U.S. ARMY.

In view of the presence of American fighting men upon the Western Front, the following particulars taken from the *United Services Gazette* will be of especial interest at the present time: The percentage of men who in normal times desire to enlist but are rejected on physical grounds is variously estimated at from 40 to 60 per cent. This means, roughly, that to obtain the 625,000 men composing the first levy it will be necessary to examine at least a million men. Under existing regulations the height must be not less than 5 ft. 4 in., nor more than 6 ft. The recruit must have good sight and hearing, and be free from signs of organic disease and from deformities of hands, feet, and ears. There must be no history of mental disorder, nor of cardiac or other lesions. In addition, he must possess at least four molar teeth. Physically, therefore, the American Army promises to be the finest in the world, more particularly at a time when much of the finest manhood of every nation has fallen on the field of battle. This should be remembered by the croakers who have said that though the financial aid of America would be invaluable, she could never render us any effective assistance in the field.

### AMERICAN RED CROSS AID FOR RUSSIA.

Our new Ally is making herself felt in many directions. Certainly not the least effective form of help is the dispatch of a special Commission to Russia, with medical supplies and surgical instruments to meet the most urgent needs. The Commission is composed of twelve men, including experts in medicine, public health, sanitation, and social service, and its purpose is not only to give immediate aid, but to study the methods whereby the American Red Cross can extend the most speedy relief to wounded soldiers and needy civilians on and behind the Russian fighting line.

### THE LATEST ORTHOPÆDIC HOSPITAL.

It is announced that a site opposite Golders Hill Park, which has a fine view over Hampstead Heath, has been taken over by the War Office. On this is to be built a hut hospital for the benefit of crippled men who have been discharged from the

Army, and whose cases are expected to respond to treatment if sufficient time is available. The institution is to be known as the Orthopædic Hospital of the Allies' Hospital Benevolent Society. It is hoped to accommodate 1,000 cases. Sir G. Wyatt Truscott is interesting himself in the matter, and donations may be sent to him at 135 New Bond Street.

### THE RED CROSS IN MESOPOTAMIA.

It is good news to hear that the Red Cross Service at Bagdad, under the charge of Major Stanley, is now in very satisfactory working order. A few hours after the victorious entry into the city by our Army, a large house was placed at the disposal of the joint societies by the Army commander, stores were brought up the Tigris as rapidly as possible, and in a week's time the depôt was in full working order. What is even more interesting is that a shipyard has been established on the banks of the river with ample facilities for turning out light craft as rapidly as they are required. The capture of Bagdad has created a new position. The Army, from being centred along a single line of river, now covers a large area of country intersected by many streams. Thus the ideal arrangement is a large fleet of river launches, picking up the wounded from certain defined stations to which they are conveyed by motor ambulances. Provision is being made for a considerable number of hospitals in Bagdad itself to avoid sending the sick and wounded the long and trying voyage down the Tigris in hot weather. An officers' convalescent hospital of fifty beds is being constructed, and funds are asked for assisting in fitting out a convalescent camp for 1,000 men. Touched as the general public have been recently by the horrors of the Mesopotamia Report, there should be no difficulty in securing well-nigh unlimited funds for this most essential work.

### DOUBLING A SATURDAY FUND COLLECTION.

A record has been created for the Grimsby and District Hospital Saturday Fund, at the last collection, when no less a sum than £1,768 was collected. Since the previous year's total was only £832, the increase is remarkable. It is largely the outcome of the popularity and the personal efforts of Alderman R. W. Roberts, J.P., the chairman of the managing committee. To double the proceeds of an annual collection in these times is a feat of which anyone may be proud, and the hospital and its secretary, Mr. Ben Chapman, must be exceedingly grateful for this result.

### EXEMPTED TUBERCULOUS PATIENTS.

Signs of the present feeling against the revision of military exemptions enactment can be seen in a resolution of the Cambridgeshire Sanatorium Benefit Sub-Committee that the Insurance Commissioners should be urged to take immediate steps in order that the instructions hitherto in force, directing recruiting officers not to call up notified cases of tuberculosis, be continued under the new Act. The Committee are of opinion that any contrary action would be a grave menace to the health of the Army and a serious danger to the public. Copies of this resolution were directed to be sent to the Insurance Commissioners, the Local Government Board, the War Office, and the National Association of Insurance Committees. It might well have been added that any person suffering from tuberculosis is of no permanent military value, excepting slight cases of lupus. The experience of most tuberculosis workers is that the familiar tendency of the disease to relapse and recrudescence is brought out almost to a certainty by any form of military service.

### CINEMA INSTRUCTION FOR ORTHOPÆDIC PATIENTS.

The development of the curative workshops for the treatment and education of orthopædic cases among the wounded has been one of the surprises and successes of the war. It is now rather a question of what activity is not undertaken than of what experiments have been tried. Through the courtesy of the West Derby Board of Guardians curative workshops have been built at Alder Hey, Liverpool, and the list of departments is an impressive one. A central smithy is surrounded by rooms for cobbling, carving, carpentry, embossed leather work, modelling in plaster, metal-turning, painting, and typewriting. Outside in the grounds gardening is undertaken. Instruction in operating the cinema is also given; a patients' band exists, and in the autumn modern-language classes are to be begun. There is also, of course, a gymnasium and a skilled staff of instructors under the supervision of Mr. Hewitt. Miss M. Townsend teaches carving and metal work, Mr. Vose the cobbling, and Mr. Dawson the carpentry. Electric and hydropathic treatment is about to be provided; a hut or shed has been obtained, and there is the nucleus of an equipment fund also. Such an array of enterprises can hardly fail of completeness. What will the next experiment be? A project is on foot to establish a similar orthopædic hospital at Manchester.

### THE WILY SHIRKER.

In a long and very interesting article the *Times* of July 9 gives an account of the various devices adopted by men wishing to escape military service. Of the numberless ruses adopted many would and must have sufficed to deceive entirely the unsuspecting medical officer. Luckily, however, modern science does not stop at physical examination, and, with the services of the analyst, bacteriologist, and alienist, there must be few who have unjustly escaped the net of military service. The gentleman suffering from acute *otitis media* who simulated the discharge with a mixture of tobacco juice and condensed milk must have been surprised when an unsympathetic board passed him into Class A; whilst one reads of others, no less resourceful, who have introduced tubercle bacilli into their sputum, or taken violent emetics prior to examination. That there are forms of fraud well-nigh impossible to detect is freely admitted. Certain drugs used to depress or accelerate the heart's action defy the most expert scrutiny. Cordite used to be taken extensively at one time by men wishing to work their discharge from the Army. But little by little these devices are becoming known to the police, as certain cases heard recently in the criminal courts go to show, and the men and their accomplices, too often, unfortunately, medical men fallen on devious ways, made to feel the weight of the law.

### THE DUBLIN MILK SUPPLY.

Professor D. Houston, of the Royal College of Science of Dublin, has sent to the Local Government Board a report on the milk supply of Dublin. From his examinations of the milk he is led to express the opinion that there is a woeful lack of cleanliness throughout the country, as well as inattention to the straining and the cooling of the milk. In many cases it is delivered in so acid a condition that it will not stand without curdling the temperature required for pasteurisation. His examinations were especially directed towards the estimation of the amount of bacterial infection present, and on the whole the milk falls below the standard of purity which is required; and some of the worst cases in this respect were examples described as "pasteurised" milk, and this is, he tells us, generally the case when the pasteurisation has been inefficiently done. A large proportion of the specimens he examined contained organisms showing that there had been contamination with dung. The presence in many cases of preservatives was shown, and though these may suffice to prevent the milk souring, so that the lactic bacilli are inhibited, yet other truly harmful germs may multiply freely. Finally, to show how widespread is the infection of milk, we may mention that when Professor Houston wished to obtain a sample of pure milk for his experimental work it took him more than a fortnight to obtain a specimen that would pass all the tests. The difficulties of obtaining a pure milk supply are enormous, for there is no other article of food which can so easily become infected as milk, and yet the importance of a full supply of good milk for the nourishment of children cannot be exaggerated.

### THE HEALTH OF MUNITION WORKERS.

Further statistical information concerning output in relation to hours of work, with special reference to the influence of Sunday labour, has been issued by the Health of Munition Workers Committee as a memorandum. The document is of a very interesting character. The conclusions arrived at, Sir George Newman explains in the introduction, are the result of careful and important investigations carried out by Dr. H. M. Vernon. In a group of nearly 100 women, kept under observation for more than a year, a considerable reduction of the hours of labour and the enforcement of Sunday rest, far from diminishing output, actually increased it. A similar result was observed when fifty-six men engaged in heavy work were studied. Dr. Vernon calls attention to facts which make it difficult to suppose that the improvement in output was a mere consequence to earn a weekly wage at least equal to that earned before. Dr. Vernon's investigations into the bearing of health on production are the more valuable in that they were undertaken in a spirit of scientific inquiry, and were not prejudiced by preconceived opinions.

### PENSIONERS WHO PLEAD POVERTY.

We are asked to state that it has been brought to the notice of the Ministry of Pensions that a number of pensioners are appealing to the charitable on the ground of insufficient means. The Local War Pensions Committee, whose address is obtainable from the nearest post office, have ample powers for dealing with all deserving cases of this nature from public funds, and the charitable public are therefore urged to refer such applicants to this body.

### SERBIANS' GRATITUDE TO AN ENGLISHWOMAN.

The Serbians have not forgotten, and do not intend to forget, Mrs. Harley, Lord French's sister, who was killed by an enemy shell at Monastir on March 7. In her memory the officers of the Serbian base have erected a monument, which was recently unveiled during an impressive full choral requiem celebrated over her grave by the Serbian clergy. The memorial, which is a solid piece of masonry, consists of blocks of grey granite, cemented together and surmounted by a white marble cross. Two marble bases bear the following inscription in gold letters in the Serbian and English languages: "To the victim of barbarians, a generous English lady, a great benefactress of the Serbian people, and a great lady. On your tomb, instead of flowers, the gratitude of the Serbs shall blossom. For your wonderful deeds your name shall be known from generation to generation.—From the Officers at a Serbian base." At the inauguration there was a large congregation of friends and admirers, including a number of British, French, and Serbian Red Cross Sisters, the deceased's daughters, and many others who had been associated with her in her noble work for Serbia.

### A FOODLESS DAY.

One of the many suggestions for the economising of the supply of food during the war is that all those members of the community who do not have to perform hard work should abstain from all food during one day in the week, and by this expedient it is hoped that sufficient food would be saved to make a material difference in the amount of the comestibles available. It is certainly true that most people, not only non-workers but workers also, do for the most part consume more food than can usefully be employed in the nourishment of the body, the excess merely being thrown away, or stored up in the body in the form of fat. Therefore, it would be possible for most people to live healthily on a reduced, and in many cases a greatly reduced diet, yet it is entirely another matter to say that anyone should be advised, even urged to fast completely from food for one whole day a week. The matter is far simpler if the abstinence is to affect only animal food, for it is not only possible, but very simple to do without animal food for even a long time, and that not only without actual harm, but in many cases with very definite benefit.

### A SCHOOL FOR ORDERLIES.

Entry into any branch of the nursing profession is not so simple a matter as it used to be. Instead of being trained to be a nurse, women are trained to be probationers, and the recent addition of schools for nurses now has its counterpart in schools for orderlies. Thus the 3rd Western General Hospital at Cardiff, of which Colonel David Hepburn is the commanding officer, had a war establishment reckoned at 520, and at present has actually 6,408 beds in use. This phenomenal expansion has led to a number of allied activities, and the institution has now opened a school for training R.A.M.C. orderlies for the Front. It would be interesting to compare the work of this school with that of the recent schools for nurses, and we hope to return to the subject in a later issue.

### ASYLUM STAFFS AND MILITARY SERVICE: "A DANGEROUS POSITION."

Mr. Guy W. Jackson, clerk to the visiting committee of the Wilts County Mental Hospital, made a remarkable statement to the Devizes Rural Tribunal when his committee appealed for the exemption of three members of the staff. Mr. Jackson said that there had been already a dangerous depletion of the staff owing to the lack of experienced and able-bodied men among the few attendants left, and the difficulty of getting men of any sort to fill the vacancies. The three men have all been several times rejected. Two are now placed in C class and one in B. The latter, who is twenty years of age, was exempted till July 30 only, the two former till the end of December. The Tribunal expressed the view that C 3 men might be left to the institution, since mental hospitals were of national importance, but that B 2 men could not be left. Mr. Jackson stated that the position with regard to the staff was felt to be so dangerous by the visiting committee that it could not accept the responsibility of having anyone to argue the merits of the cases lest it should be said that the argument used was not well enough put to retain the men.

### DENTAL STUDENTS AT GUY'S HOSPITAL.

The military representative at the Southwark Tribunal cast a serious aspersion on the governors and administrators at Guy's Hospital when Dr. W. S. Pembrey applied for the exemption of two dental house surgeons. Both of these were young men who had been passed for military service, and had qualified as dental surgeons. The authorities at Guy's desired to retain their services in order that they might keep open the third part of their dental department, the rest having had to be closed down through shortage of staff. If these two were taken, there was a danger that the whole of the department would have to be closed. It was suggested that older men might be engaged to fill these posts, but, as Dr. Pembrey reasonably pointed out, they were unable to pay the extraordinarily large fees now demanded by dental surgeons ineligible for military service, and consequently had to depend on younger men for the maintenance of something approaching a competent dental staff.

### THE MILITARY COMPLAINT.

The complaint of the military representative was that an undertaking had been given that when all the students at the hospital had passed their final examinations they should, "willy nilly," go into the Army. The Tribunal, however, who know the needs of the district, and are cognisant of the great boon the hospital is to the poor of South London, thought that some staff should be retained to cope with the needs of the civil population, especially in case of air-raids, and decided to grant the two dental surgeons three months' exemption each on condition that they remained at Guy's. The military representative thereupon made the caustic remark that "the best way to keep out of the Army was to become a dental student at Guy's in 1914," a remark which was most strenuously and justly resented by Dr. Pembrey. Those who realise the great sacrifices that Guy's men have made since the outbreak of war will associate themselves with Dr. Pembrey in his refutation of the aspersion cast upon the hospital and its staff. Reference to the "War Service List" compiled by the *Guy's Hospital Gazette* shows that an enormous number of Guy's men are serving with the Navy or Army, both as combatants and as medical officers, and that they have gained many distinctions. As showing the extreme difficulty of maintaining the great volume of work with a dearth of staff it is only necessary to refer to the last annual report of the hospital, which states that before the war the permanent staff numbered fifty, and the residential staff twenty, which have now been reduced to twenty-eight permanent staff and seven residential staff. These figures show that the governors are perfectly justified in trying to retain the services of two dental house surgeons in order that a small part of the dental department may remain open so that the very worst cases may be treated.

### THIS WEEK'S DRUG MARKET.

The drug market has been rather quiet this week, with few features of particular importance. There has been more activity in quinine, and a few parcels have changed hands at a rather higher price; in spite of the reduced stocks, however, it is not anticipated that values will rise substantially in view of the attitude of the Government. Potassium bromide is about sixpence per pound dearer, and one maker has added more than this to his quotation. Saccharin is in good demand at high rates. English refiners of camphor are much behind with their deliveries, and are inclined to refuse new orders. There is still a big demand for glycerophosphate of potash and calcium, which are rather scarce; other glycerophosphates are more readily obtainable. Belladonna root is very scarce. Senega root is rather dearer. Higher prices are asked for cassia oil, aniseed oil, clove oil, sarsaparilla, calumba root, and gentian root. In synthetic drugs there are practically no changes, and there has been no further rise in the prices of salicylates.

